# Observing the devastating coffee berry borer (*Hypothenemus hampei*) inside the coffee berry using micro-computed tomography

**DOI:** 10.1038/s41598-018-35324-4

**Published:** 2018-11-19

**Authors:** Ignacio Alba-Alejandre, Javier Alba-Tercedor, Fernando E. Vega

**Affiliations:** 1 Department of Zoology, Faculty of Sciences, University of Granada, Campus de Fuentenueva, 18071 Granada, Spain; 2 Sustainable Perennial Crops Laboratory, United States Department of Agriculture, Agricultural Research Service, Beltsville, MD 20705 USA

## Abstract

The coffee berry borer is the most devastating insect pest of coffee throughout the world. The insect spends most of its life cycle inside the coffee berry, which makes it quite difficult to observe its behaviour. Micro-computed tomography (micro-CT) was used to observe all developmental stages of the coffee berry borer inside coffee berries (*Coffea canephora*). An interesting oviposition pattern involving a sequential placement of eggs starting in the periphery of the seed and moving inwards was observed. Micro-CT should be useful in elucidating unknown life history aspects of other seed-feeding bark beetles as well as of bark and ambrosia beetles in general.

## Introduction

Two coffee species constitute the bulk of coffee trade worldwide: *Coffea arabica* L. and *Coffea canephora* Pierre ex. A. Froehner (also known as robusta coffee). Coffee production is an important source of employment and revenue for coffee-producing countries and the 2012 estimated value for the worldwide coffee industry was US$173 billion^1^. A major constraint to coffee production is the damage caused by insect pests and plant pathogens^2^. Even though many insect species have been reported attacking coffee plants^3^, only one insect, the coffee berry borer (*Hypothenemus hampei* (Ferrari); Coleoptera: Curculionidae: Scolytinae), has specialized on consuming and reproducing in the seeds inside the berry, making it the most economically important insect pest of coffee worldwide^4^.

The coffee berry borer is a cryptic insect, i.e., females spend most of their life cycle inside coffee berries and males are born and die inside the berry. This particular aspect of the insect’s biology makes it difficult to study its behaviour and quite difficult to control. The life cycle begins when an adult female, referred to as the colonizing female, emerges from an infested berry and bores a hole into another berry, usually through an area known as the disc, which was originally the floral disc of the flower. The female enters the seed and builds galleries, where she oviposits. The emerging larvae consume the seed and once females reach the adult stage, they mate with their siblings^4^. Males are smaller than females, have vestigial wings and rudimentary eyes, and as mentioned above, they never leave the berry^4^. Their main role in life is mating.

A recently-developed technique useful in studying the behaviour of the coffee berry borer involves artificial diet sandwiches, consisting of artificial coffee berry borer diet^5^ placed between two glass plates, followed by introducing insects and sealing the plates^6^. This technique has shown that the insect exhibits subsocial behaviour^6^. Because observing the development and behaviour of the insect in intact berries is impossible, the use of an X-ray-based technology known as micro-computed tomography (micro-CT) is ideal to learn more about the insect’s life history inside the berry. The first high resolution CT study on plants was published in 2003^7^ and in the intervening 15 years the technique has become frequently used in plant and insect-related studies. Micro-CT has also been used to observe the coffee bean weevil (*Araecerus fasciculatus* (De Geer); Coleoptera: Anthribidae) inside the coffee berry^8^. This insect has been reported inside coffee berries on a handful of occasions and is best known as a stored product pest of many agricultural products^8^. In addition, a preliminary micro-CT report of coffee berry borers inside the berry has been published^9^.

In this paper, we present results of observations of two coffee berry borer-infested coffee berries using the micro-CT technique. In one of the berries (berry 1) we observed several developmental stages while the second berry (berry 2) had just been colonized by an adult female, and only two batches of eggs had been oviposited.

## Results

**Figure 1:**
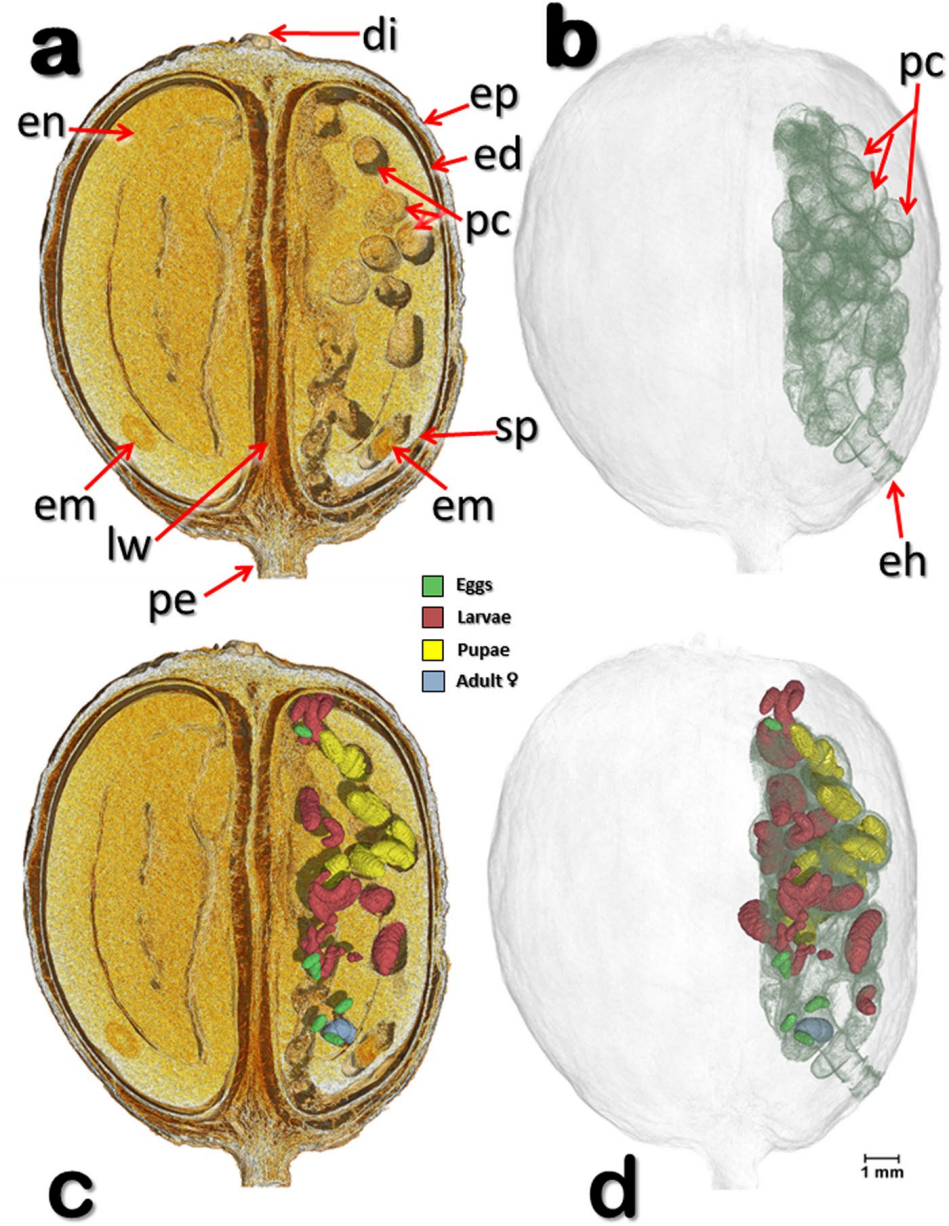
Volume rendered images of a coffee berry bored-infested coffee berry (berry 1). Meso-sagittal section at the broadest plane, perpendicular to the locule wall, showing the two seeds and different components of the berry (**a**), and the various developmental stages of the insect (**c**). Note than only one of the two seeds is being used by the insects (**a**,**c**). Insects have been digitally removed to show details of the insect galleries and pupal cells (**a**,**b**) as well as the entrance hole bored by the colonizing female (**b**). Berry and seed components have been digitally removed to reveal the specific location of the different development stages of the insects (**d**). Abbreviations: di = disc (style remnant); ed = endocarp (parchment); eh = entrance hole; em = coffee embryo; en = endosperm (coffee seed); ep = epicarp (outer skin); lw = locule wall (ovary); pc = pupal cell; pe = pedicel; sp = spermoderm (silverskin).

**Figure 2:**
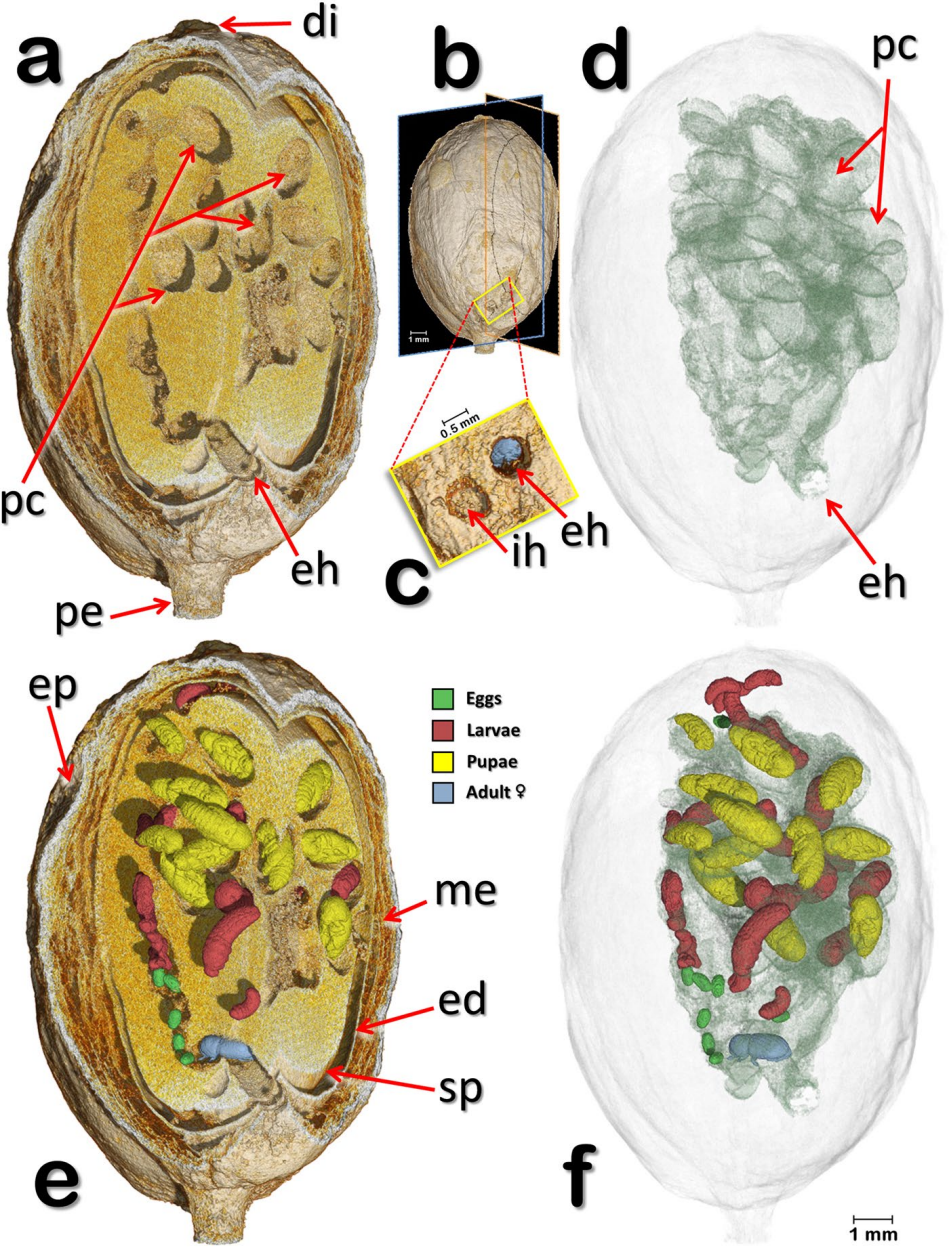
Volume rendered images of a coffee berry bored-infested berry (berry 1). Oblique-lateral and oblique-apical section views (**a**,**e**), as shown in (**b**). The insects have been digitally removed to show details of the seed, galleries, and pupal cells (**a**,**d**). Detail of the latero-basal surface of the coffee berry showing an incomplete hole and the entrance hole bored by the colonizing female, whose posterior can be seen inside the gallery (**c**). Developmental stages of the insect (eggs, larvae, pupae, and adult colonizing female) in different colors (**e**,**f**). The coffee seed and components of the berry have been made transparent to show the galleries and the specific location of the different developmental stages of the insect (**f**). Abbreviations: di = disc (style remnant); ed = endocarp (parchment); eh = entrance (penetration hole); ep = epicarp (outer skin); ih = incomplete hole; me = mesocarp (mucilage); pc = pupal cell; pe = pedicel; sp = spermoderm (silverskin).

**Figure 3:**
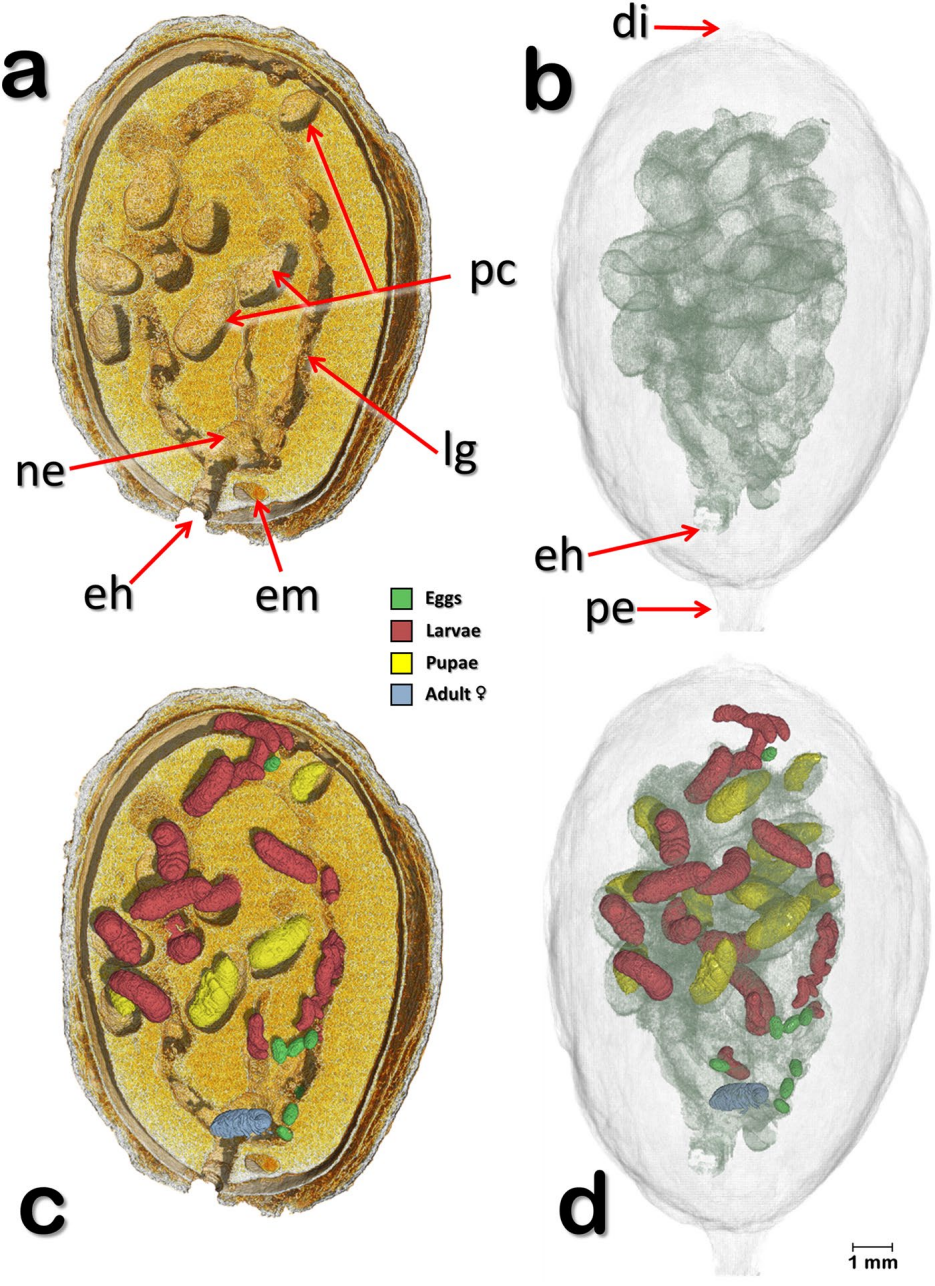
Volume rendered images of a meso-sagittal section (**a**,**c**) through an infested coffee berry (berry 1) sectioned at the side opposite to that shown in Fig. 1. The insects have been digitally removed to show details of the galleries and the pupal cells (**a**,**b**). Developmental stages of the insect (eggs, larvae, pupae, and adult colonizing female) have been separately segmented to unveil their location inside the berry (**c**,**d**). Abbreviations: di = disc; eh = entrance hole; em = coffee embryo; lg = larval gallery; ne = nest; pc = pupal cell; pe = pedicel.

Figures 1–3 show different views of one of two coffee berry borer-infested coffee berries (berry 1) observed with micro-CT technology. Several components of the berry have been identified, including the disc, pedicel, endocarp (parchment), mesocarp (mucilage), spermoderm (silver skin), locule wall (ovary), and coffee embryo.

Figure 1a (berry 1) depicts a meso-sagittal cut of a coffee berry showing the two coffee seeds separated by the locule wall, as well as the galleries and pupal cells created by the insect. We use the term “pupal cell” as used by Hopkins^10^, Swaine^11^, Dodge^12^, and Browne^13^, to describe the enclosed chamber where pupation occurs (Figs 1–3). According to Hopkins^10^, “The pupal cell is formed by the prepupal larva or by the pupa itself and is usually located at the end of the larval mine or food burrow of the larva.” The pupal cells observed inside the coffee berry were formed by the second larval instar, a developmental stage that includes the prepupa^14^. This observation agrees with Bergamin^15^, who stated the coffee berry borer prepupa is responsible for building the pupal cell.

Figures 1–3 (berry 1) show different section planes through a coffee berry with the location of the different development stages, including the colonizing female. The coffee berry has been made transparent (Fig. 1b) to focus on the insect galleries and pupal cells and on the entrance hole, which is the area through which the coffee berry borer enters the berry, although in this berry the insect entered through the side of the berry, close to the petiole. Figure 1c depicts a meso-sagittal cut that includes the developmental stages of the insect in different colors, with the components of the berry digitally removed in Fig. 1d to focus on the distribution of the insects within the seed. It is important to note that only one seed has been infested by the insects.

Figure 2 (berry 1) shows oblique-lateral and oblique-apical sectional views. In Fig. 2a, the insects have been digitally removed to focus on the galleries, pupal cells, and the entrance hole. A detail of the entrance hole with the colonizing female in blue is shown in Fig. 2c, next to an incomplete hole. In Fig. 2d,f the coffee berry and its components have been made transparent to focus on the entrance hole, galleries, pupal cells (Fig. 2d) and on the different development stages (Fig. 2f). The colonizing female has moved throughout various areas of the seed, ovipositing in an interesting pattern, discussed below.

Figure 3 (berry 1) is a meso-sagittal cut depicting a volume rendered coffee berry bored-infested coffee berry cut at the opposite side of the berry shown in Fig. 1. In Fig. 3b,d the coffee berry and its components have been made transparent to focus on the galleries bored by the insect (Fig. 3b) and on the different developmental stages of the insect (Fig. 3d). In Fig. 3a,b the insects have been removed by software to show details of the galleries. Figure 3c shows the different development stages of the insect within the galleries and pupal cells.

**Figure 4:**
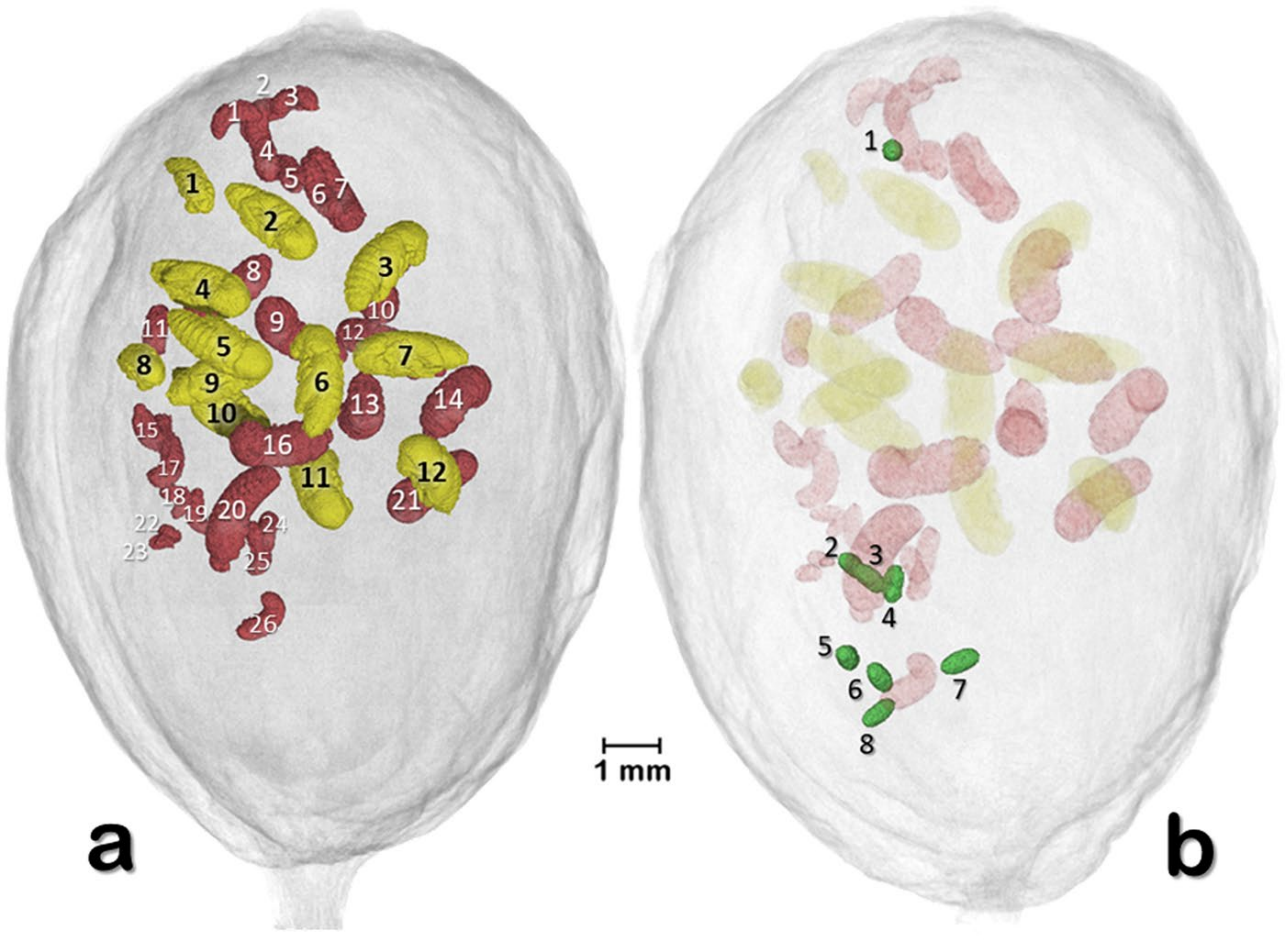
Volume rendered images of 26 larva and 12 pupae (**a**) and eight eggs (**b**) (berry 1). Each one has been individually identified. Pupa #1 is a male pupa. Eggs in (**b**) were oviposited in an area of the seed behind the larvae and pupae.

**Figure 5:**
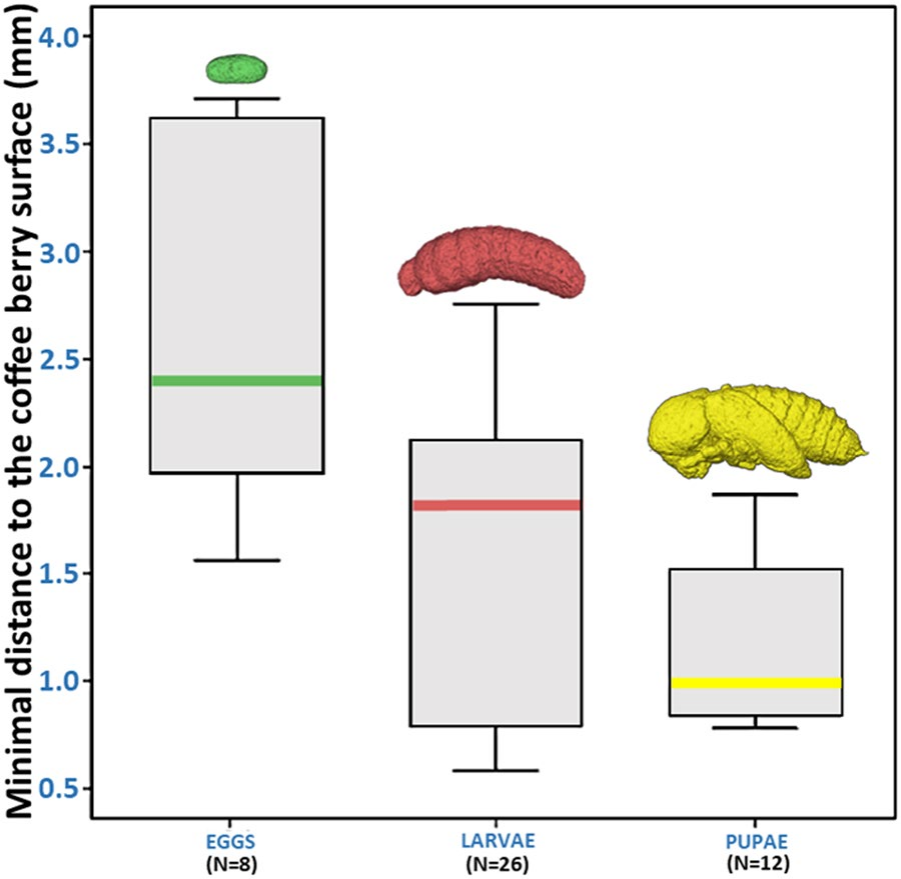
Minimal distance of eggs, larvae and pupae to the surface of the coffee berry (berry 1). Eggs were further away from the berry surface, followed by larvae and pupae. Differences in location were significantly different based on Kruskwal-Wallis test (p = 0.004).

Figure 4 (berry 1) presents two volume rendered images of 26 larva, 12 pupae, and eight eggs, with each one individually identified. This image and Supplementary Video S1 revealed that the placement of the three developmental stages appeared to have a pattern. The minimal distance (mm) of the eggs, larvae, and pupae to the surface of the berry is presented in Fig. 5. The location of each developmental stages was significantly different based on a Kruskal-Wallis test (chi-square 11.009, df = 2, p = 0.004), with eggs being further from the surface, followed by larvae, and pupae (means: 2.70, 1.83, and 1.25 mm, respectively).

**Figure 6:**
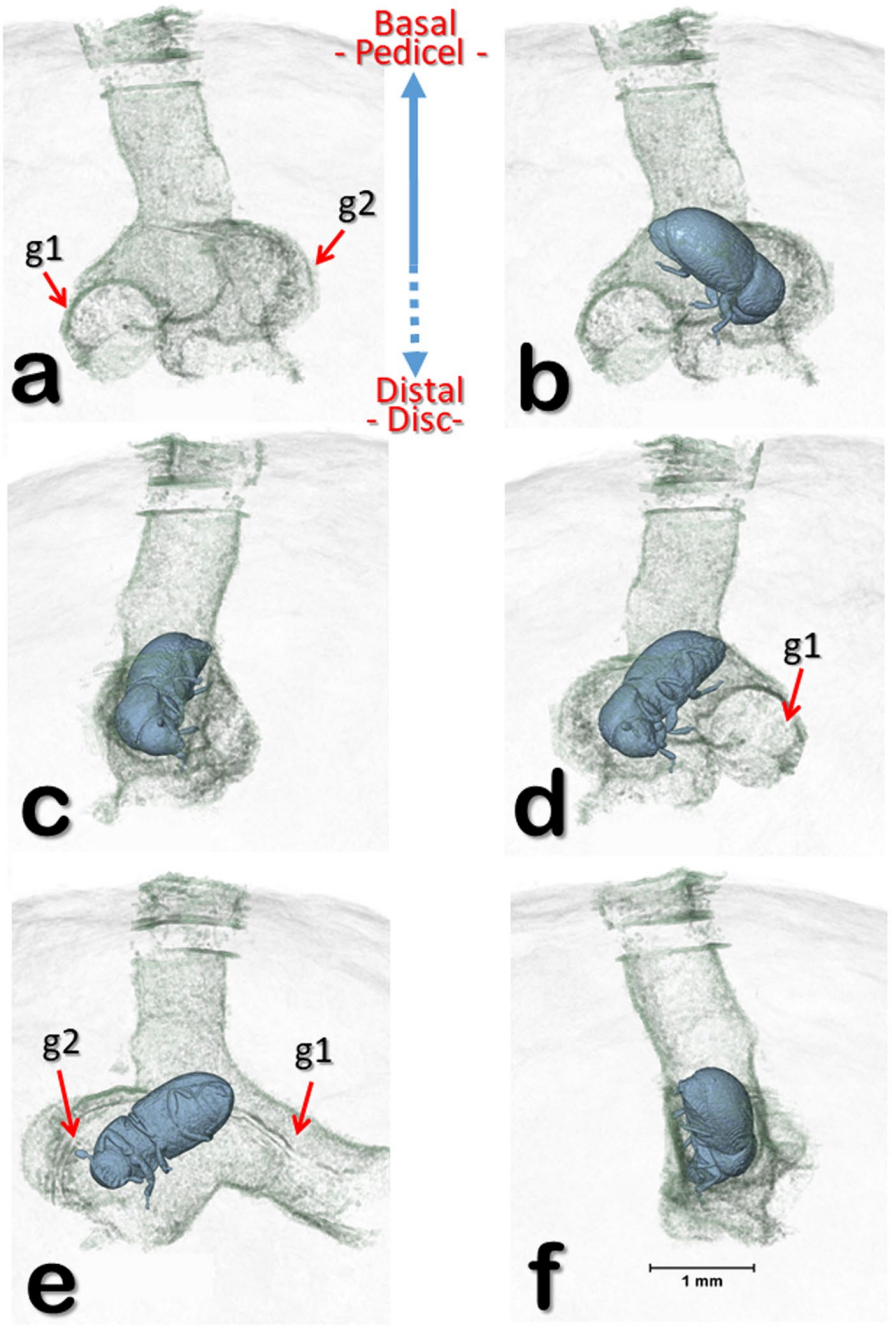
Volume rendered image reconstructions of the entrance hole and bifurcating galleries (berry 1) at different rotation angles (**a**–**f**). The insect has been digitally removed to show the bifurcating galleries (**a**). The eggs and galleries shown in Figs 1–3 have been digitally removed to focus on the area at the end of the entrance hole. Abbreviations: g1 = 1^st^ gallery; g2 = 2^nd^ gallery.

**Figure 7:**
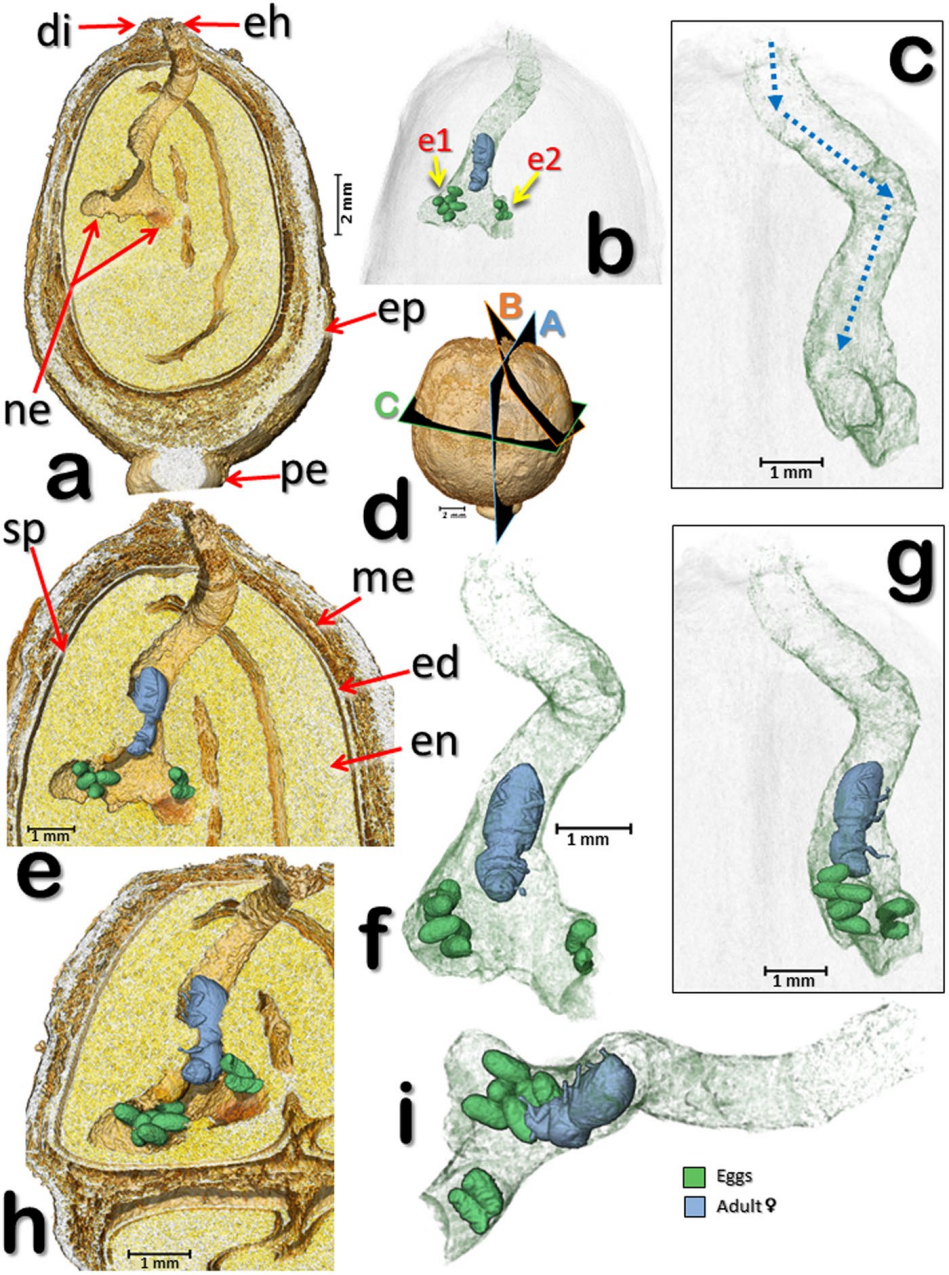
Volume rendered images of a coffee berry (berry 2) in the initial stages of colonization by a colonizing female. Lateral sections (**a**,**e**,**h**) according to planes “A”, “B”, and “C” shown in (**d**). The female and eggs have been digitally removed to show the nest and initial gallery following the entrance hole (**a**). The components of the berry have been digitally removed to focus on the entrance hole, gallery, colonizing female, and two batches of eggs (**b**). The female adult and eggs have been digitally removed and the gallery rotated 90° (**c**) in relation to views in (**a**) and (**b**), with the adult and eggs back in place (**g**). The dotted blue arrows (**c**) are helpful in visualizing the alternating angles in the gallery following the entrance hole, and at the end, two nests with eggs (**a**,**b**,**e**–**i**). Detail of the female and eggs inside the galleries and cavities (**f**); visualization has been rotated 45° in relation to (**a**); 45° rotation from the petiole, in a distal view (**i**). Abbreviations: di = disc (style remnant); e1 = 1^st^ egg batch; e2 = 2^nd^ egg batch; ed = endocarp (parchment); eh = entrance hole; en = endosperm (seed); ep = epicarp (outer skin); me = mesocarp (mucilage); ne = nest; pe = pedicel; sp = spermoderm (silverskin).

Figure 6 (berry 1) is a volume rendered image reconstruction of the entrance hole bored by the colonizing female shown in the previous figures, with two bifurcating galleries at different rotation angles (b-f). In Fig. 6a, the insect has been digitally removed to show details of the bifurcating galleries. A similar pattern in a different berry (berry 2) is shown in Fig. 7 and Supplementary Video S2, which depicts a second coffee berry borer-infested coffee berry (berry 2) observed with micro-CT. This berry had been recently infested by a colonizing female based on the presence of eggs and no larvae or pupae. The two egg batches reveal an oviposition pattern similar to that reported for the first berry (Figs 4 and 5). In this case (Fig. 7; berry 2), one egg batch is 2.0 mm from the surface of the berry (hypothetically, this would be the first batch of eggs) and the other batch is 3.7 mm (hypothetically, the second batch), respectively.

## Discussion

Several bark beetle species from different genera are known to attack seeds, among these, *Araptus*, *Coccotrypes*, *Hypothenemus*, *Pagiocerus*, and *Scolytodes*^13,16,17^. Not much is known about the life history of these bark beetle species inside the seed, including the distribution of eggs as well as the shape of the galleries.

Our visualization of coffee berry borers inside the coffee berry using micro-CT reveals the advantages of using this technology to increase our knowledge on the life history of the insect. For example, the distribution of the developmental stages of the insect at different distances from the surface of the berry suggests that the colonizing females starts ovipositing close to the seed periphery and oviposition then moves inwards, towards the center of the seed. This might be a strategy to disperse the progeny in order to reduce competition. It is important to emphasize that this is purely speculative at this moment and that additional micro-CT observations of coffee berry borer-infested berries are needed to determine if this oviposition pattern holds.

Figures 1–3 show that the insects are only feeding on one of the two coffee seeds, although as the insect population increases, it can be expected for insects as well as the colonizing female to move to the second seed based on the extremely high number of eggs that can be found inside one berry, e.g., 164^18^ and 288^19^. At the time of analysis, the berry depicted in Figs 1–3 contained 47 insects, including the colonizing female.

Of the various developmental stages shown in Fig. 4, there are eight unhatched eggs (Fig. 4b, Supplementary Video S1) that were oviposited in an area of the seed that is opposite the original batch of eggs resulting in the larvae and pupae seen in Fig. 4a. One of the eggs is in the distal area of the berry, close to the disc (Figs 1–4). This reveals that the colonizing female does not necessarily place all the eggs in one location. Even though we don’t know if these eight eggs would end-up hatching, it is safe to state that there was 100% hatching in the two previous egg batches resulting in 26 larvae and 12 pupae, as there are no eggs unaccounted for. There are no reports assessing egg fertility inside coffee berries as dissection of a berry with subsequent assessment of hatchability might not be reliable due to the drastic changes in microhabitat experienced by the dissected eggs. Therefore, this is another area that can be further explored using micro-CT.

The two egg batches in Fig. 7 resemble Browne’s^13^ description of *Coccotrypes*, which as mentioned above, also feeds on seeds: “In fruit and seeds the eggs are placed at the end of a short tunnel, and the larvae enlarge this and also make other irregular tunnels and chambers in all directions.” Figure 7 also depicts alternating angles in the gallery following the entrance hole.

Adult coffee berry borer females emerge from the berry using the entrance hole built by the colonizing female. Similarly, “In *Anisandrus* and *Xyleborus*… the brood matures and emerges by way of the entrance gallery”^12^. This is interesting because there is a highway of tunnels and cavities that the new adults have to transit through in order to find the entrance hole, although it is possible that they might use visual cues (e.g., light) and air currents to locate it.

Several papers describe the different types of galleries created by bark beetles^10–12,20,21^. Hopkins^10^ states that even though different bark beetle genera can make the same or similar type of gallery, “galleries are of taxonomic importance” and the same insect species will construct the same or similar type of gallery in different hosts, thus the shape of the gallery is not determined by the host plant. There are no studies focused on gallery formation by seed boring bark beetles; this fact presents an opportunity to use micro-CT to study gallery form and function in seeds.

As a footnote, in a proceedings paper presenting our preliminary results^9^, we had stated that a female could be seen grooming an egg. After more detailed analysis including visualization at different angles, it became evident that the female was not grooming the egg and instead had her mouthparts in the proximity of rasped seed material.

In conclusion, the use of micro-CT techniques to study the coffee berry borer has resulted in interesting observations. Future research will expand on these observations to determine if they are the norm. Micro-CT could also be useful for learning more about the cryptic life of other bark beetles, including ambrosia beetles.

## Methods

### Coffee berries

J.A.T. collected coffee berries (*Coffea canephora* Pierre ex. A. Froehner; Rubiaceae) at a coffee plantation in Vietnam (*Me Linh Coffee Garden*; 11°53′57.39′′ N, 108°20′51.16′′ E; 1043 masl) in November 2017. Out of 55 *C*. *canephora* berries collected in the field, 12 (22%) were infested with the coffee berry borer. Seven berries (13%) had the insect entrance hole on the disc or very close to it. Five berries (9%) had the entrance hole in the petiole area: two were at a 45° angle through the petiole, and three were next to the petiole. None of the berries had more than one entrance hole and only one had an incomplete hole next to a complete entrance hole.

### Micro-CT scans

The berries were kept at room temperature in the laboratory at the University of Granada and were mounted on Basotect^®^ (a light weight melamine foam manufactured by BASF) placed inside a plastic container. A few drops of ethyl acetate were placed on the Basotect^®^ and the plastic container was closed. The emanating vapors produced by the chemical quickly immobilize and kill the insect. Following a 30 min period, micro-CT observations were initiated using a Bruker SkyScan 1172 microtomograph (Bruker-micro CT, Kontich, Belgium) with a Hamamatsu L702 X-ray source and a Ximea 11 megapixels camera. The setting parameters were as follows: voltage = 48 KV; current = 124 µA; isotropic voxel size = 3.25 µm (except for Fig. 7 and Supplementary Video S2, which were 6.7 µm); image rotation step = 0.3° (except for Fig. 7 and Supplementary Video S2, which were 0.2°); 360° of rotation scan, and an Al 0.5 mm filter, resulting in two overlapping connected scans with scan durations of 2 h:20 min:40 s for berry 1 and 1 h:24 min:58 s for berry 2. The most recent versions of the Bruker micro-CT’s Skyscan software (NRecon, DataViewer, CTAnalyser) were used for primary reconstructions and the “cleaning” process to obtain the datasets of “slices” as previously described^22^. Volume rendered images and videos were obtained with FEI’s Amira’s software v. 6.5.0^23^ (Thermo Fisher Scientific, Waltham, MA).

## Electronic supplementary material

Supplementary Information

Supplementary Video S1

Supplementary Video S2

## Data Availability

The datasets generated and analyzed during the course of the study are available from J.A.T. upon reasonable request.
