# Supplementary material for: Observing the devastating coffee berry borer (Hypothenemus hampei) inside the coffee berry using micro-computed tomography
Source: Sci Rep. 2018 Nov 19;8:17033. doi: 10.1038/s41598-018-35324-4 (PMC6242942; doi:10.1038/s41598-018-35324-4)
Supplement: Supplementary file 1 — Supplementary Information [file 41598_2018_35324_MOESM1_ESM.docx]

**Supplementary Information**

**Observing the devastating coffee berry borer (*Hypothenemus hampei*) inside the coffee berry using micro-computed tomography**

Ignacio Alba-Alejandre^1^, Javier Alba-Tercedor^1^, and Fernando E. Vega^2,*^

^1^Department of Zoology, Faculty of Sciences, University of Granada, Campus de Fuentenueva, 18071-Granada, Spain

^2^Sustainable Perennial Crops Laboratory, United States Department of Agriculture, Agricultural Research Service, Beltsville, MD, 20705, USA

^*^ Correspondence and requests for materials should be addressed to F.E.V. (email: [Fernando.Vega@ars.usda.gov](mailto:Fernando.Vega@ars.usda.gov)) or J.A.T. (email: [jalba@ugr.es](mailto:jalba@ugr.es))

**Supplementary Videos (mp4)**

**Supplementary Video S1**. Developmental stages (eggs, larvae, pupae, and adult colonizing female) of the coffee berry borer inside a coffee berry (berry 1). On the left side the developmental stages of the insect and the berry have been digitally removed to focus on the galleries and pupal cells. On the right side, the distribution of the different developmental stages can be observed.

**Supplementary Video S2**. Coffee berry (berry 2) with entrance hole and gallery built by a colonizing female, ending in bifurcating nests with eggs. Left: entrance hole, gallery and nests with female and eggs removed. Right: Colonizing female and eggs in place. Note that the entrance hole and gallery are not built in a straight line.
